# A Case of Eclampsia Parturientium

**Published:** 1850-03

**Authors:** David Hutchinson

**Affiliations:** Mooresville, Ind.


### ARTICLE III.

*A Case of Eclampsia Parturientium.*---By DAVID HUTCHINSON, M.D., of Mooresville, Ind.

Nov. 15th, 1849, 10 o'clock A. M. Was called to Mrs. S. B.; aet. 21; first pregnancy; at the completion of the ninth month. Face, upper and lower extremeties œdematous.

Labia pudenda swollen; each labium as large externally as a man's hand; pulse full, not very frequent; suffers from soreness of swollen labia. Prescribed a cathartic of Pulv. Jalap and Bitartarate of Potass.

Five o'clock, P. M. Was called again. Pulse full, tense and frequent; has complained of being blind; head was hot, and takes but little notice of any thing. Venesection, 20 ℥ before the operation was complete a paroxysm of convulsions ensued, which lasted between five and ten minutes. The muscles of the face and limbs were violently contorted. Frothing at the mouth, and dilated pupils, followed by insensibility and stertorous breathing. Cold applications were assiduously applied to the head, and sinapisms to the calves of the legs and nape of the neck. At this period, the face was flushed, head hot, pulse 140 and 150 per minute. In twenty minutes she had another paroxysm of convulsions, more violent than the first. Pulse very small and frequent in the paroxysm; head hot; frothing at the mouth, and stertorous breathing and insensibility; jaws clenched; cannot swallow; poured cold water in a stream from a pitcher on the head; examined per vaginam and found the os uteri dilated enough to admit the point of the fore finger. It is perhaps worthy of remark, that at the accession of the convulsions, the face became livid; and the pulse small and imperceptible, showing a great determination of the blood to the internal organs, and in the interval between the paroxysms, the face was flushed, and the pulse full and frequent. In twenty minutes from the last paroxysm, a third came on, with all the former distressing symptoms. Cold applications were still applied to the head, and I now determined to bleed again; but while I was preparing for the operation, a fourth paroxysm ensued. I opened a vein and bled to 24 ℥, and poured cold water on the head. I now hoped that the convulsions had ceased, but in three quarters of an hour, she had a fifth paroxysm; although not so severe, nor of so long duration as the former, from the bleeding and the energetic application of cold water to the head, the temperature of the head was reduced; and the pulse was also reduced in fulness and frequency. In about two hours from the last paroxysm, she looked around and recognized those that were about her from their voices; said she could not see. The five paroxysms occupied two hours. As there had not been any operation from the cathartic taken in the morning, and as she was now able to swallow I gave her a cathartic of of Calomel and Jalap a a x grs., but the stomach rejected it. I gave her Salts and Senna, but the stomach still rejected medicine, and I permitted her to rest till morning.

Nov. 16th, 10 o'clock, A. M. She has had no return of convulsions; stomach still irritable and rejects medicine; pulse frequent; not so tense; no labor pains. Prescribed 20 grs. Calomel.

One o'clock, P. M. She has not vomited the calomel; has labor pains; labia pudenda very much swollen; head of the fœtus resting on the floor of the pelvis; no return of convulsions. Her labor proceeded regularly, and she was delivered of a living female child at 5 o'clock P. M. On the expulsion of the child, another paroxysm of convulsions ensued, which was not as severe, nor of as long duration as the others.—The placenta was expelled in a few minutes, and sensibility soon retured. Cold applications were kept applied to the head, and during the night the calomel produced three evacuations from the bowels.

Nov. 17th, 10 o'clock A. M. She has had no return of convulsions; severe febrile irritation; pulse too frequent; general œdema of the body. Gave Salts and Senna until free purging is produced. Suffice it to say, that by moderate purging with Jalap and Bitartrate of Potass, the œdema disappeared, and with exception of inflammation of the mamma, she recovered in a reasonable time.

I think that the condition of the system, predisposing to an attack of convulsions, in the last stage of pregnancy appears to depend on a variety of circumstances, viz: an increased quantity of blood in the circulating system, and the mechanical pressure of the gravid uterus, on the large blood-vessels situated within the abdomen, producing irregularity in the circulation; and also an exalted sensibility of the nervous system, rendering it very susceptible of morbid impressions; together with great distension of the abdomen and œdematous swelling of the face; and in the above case, irritation from swelling of the labia pudenda, produced by the position of the child in the pelvis. I think it is not unreasonable that those conditions existing would bring about a determination of blood to the brain, and convulsions, although I know that uterine irritation is generally assigned as the predisposing cause; yet I am satisfied that other predisposing causes must also exist, but whatever may be the predisposing causes, the brain is the organ that principally suffers; and when death takes place, bloody and serous effusions are discovered in the cranium,—there is an efflux of blood to the brain; hence the necessity for active bloodletting; and I think the above case a good example of the value of bloodletting. She is a weakly and delicate female, and under ordinary circumstances, would not bear bloodletting to any great extent; yet 44 ℥ was abstracted in less than two hours, without the system appearing to feel the loss of blood. In puerperal convulsions, there exists what Marshall Hall terms a tolerance of the loss of blood.

